# Relationship between Metabolic Syndrome Components and COVID-19 Disease Severity in Hospitalized Patients: A Pilot Study

**DOI:** 10.1155/2022/9682032

**Published:** 2022-08-24

**Authors:** Hande Erman, Banu Boyuk, Meltem Sertbas, Ali Ozdemir

**Affiliations:** ^1^Department of Internal Medicine, University of Health Science, Kartal Dr Lutfi Kirdar City Hospital, Istanbul, Turkey; ^2^Department of Internal Medicine, University of Health Science, Fatih Sultan Mehmet Education and Research Hospital, Istanbul, Turkey

## Abstract

**Background:**

Preliminary data suggest that patients with comorbidities are more susceptible to severe COVID-19 infection. However, data regarding the presence of metabolic syndrome (MetS) in patients with COVID-19 are scarce.

**Aim:**

In the present study, we aim to investigate the association between MetS components and disease severity in hospitalized COVID-19 patients.

**Methods:**

We conducted a prospective observational study of 90 hospitalized patients with COVID-19 pneumonia at a tertiary hospital. The study population consisted of inpatients who tested positive by the reverse transcription polymerase chain reaction (RT-PCR) for SARS-CoV-2. Patients with critical COVID-19 disease on admission were excluded. Adult Treatment Panel III of the National Cholesterol Education Program (NCEP-ATP III) criteria were used to define MetS. Laboratory analysis and thorax CT were performed on admission.

**Results:**

90 patients, 60 moderate and 30 severe COVID-19 patients, included in the study. The percentage of MetS cases was higher among severe COVID-19 patients (*p*=0.018). Of the MetS criteria fasting blood glucose (*p*=0.004), triglycerides (*p*=0.007) were significantly higher in patients with severe COVID-19 disease with no statistical significance found in waist circumference (WC) (*p*=0.348), systolic blood pressure (*p*=0.429), and HDL-C levels (*p*=0.263) between two groups. Body mass index (BMI) values were similar in both severe and moderate cases (*p*=0.854). In logistic regression analysis, serum triglycerides (*p*=0.024), HDL-C (*p*=0.006), and WC (*p*=0.004) were found as independent prognostic factor for severe COVID-19 infection.

**Conclusion:**

Severe COVID-19 patients have higher rates of MetS. Serum triglycerides, HDL-C, and WC have an impact on disease severity in COVID-19.

## 1. Introduction

The coronavirus disease 2019 (COVID-19), caused by SARS-CoV-2, reached 446.511.318 confirmed cases, according to the report published by the World Health Organization (WHO) on March 9, 2022. COVID-19 has resulted in mortality in over 6.004.421 cases by now [1]. COVID-19 infection has variable clinical presentation, which varies from asymptomatic patients to severe cases of respiratory failure [1]. Although COVID-19 disease primarily affects the respiratory system, preliminary reports revealed that the highest mortality is belong to patients with cardiovascular disease (10.5%), diabetes mellitus (7.3%), chronic respiratory disease (6.3%), hypertension (6.0%), and cancer (5.6%) [2]. Several studies underline the link between increased mortality in COVID-19 disease and impaired metabolic health that is characterized hyperglycemia, hypertension, and dyslipidemia [3]. However, there are limited data about the associations between COVID-19 disease and metabolic syndrome components.

Metabolic syndrome (MetS) is important in terms of developing cardiovascular events and related deaths [4]. Estimated worldwide prevalence of MetS is %25 [5], suggesting the clinical importance of identifying possible effects of MetS on COVID-19 disease course. Limited reports demonstrated increased risk of adverse and fatal outcomes in COVID-19 patients with MetS [6,7]. In this prospective study, we aimed to clarify the effects of MetS and its components on COVID-19 disease severity in moderate and severe cases.

## 2. Methods

### 2.1. Study Design and Participants

In this cross-sectional, observational, prospective study from 120 patients hospitalized with COVID-19 disease, 60 (36 male and 24 female) moderate COVID-19 patients and 30 (24 male and 6 female) severe COVID-19 patients were included. Patients unable to stand upright due to respiratory distress (*n* = 20), acute renal failure (*n* = 4), chronic renal failure (*n* = 3), hyperthyroidism (*n* = 1), hypothyroidism (*n* = 2), and Cushing disease (*n* = 1) were excluded for analysis. Patients were diagnosed with COVID-19 pneumonia with mild-moderate and severe based on previously described clinical severity criteria [8]. According to the accepted criteria, mild cases have minor clinical symptoms without sign of pneumonia on imaging; moderate cases have fever and respiratory symptoms with radiological findings of pneumonia; severe cases have respiratory distress (≥30 breaths/min) or oxygen saturation (≤93%) at rest [8]. Moderate, severe, and critical COVID-19 cases were followed on ward; therefore, the study group did not contain mild cases. In addition, patients with critical COVID-19 (i: respiratory failure and requiring mechanical ventilation, ii: shock, and iii: with other organ failure that requires ICU care) at admission and in the ward were excluded due to inability to perform anthropometric measurements. The exclusion criteria were the presence of chronic and acute kidney disease, hypothyroidism, hyperthyroidism, and Cushing disease. MetS was defined with American Heart Association and National Heart, Lung, and Blood Institute criteria [9]. The study was conducted in accordance with the Turkish Ministry of Health and the Helsinki Declaration (Date: 10.06.2021, number: 2021/66) after gaining approval from the University of Health Science Fatih Sultan Mehmet Education and Research Hospital Ethics Committee. Informed consent was taken from all the participants.

### 2.2. Definitions

The presence of MetS was defined as the presence of any three of the following criteria according to American Heart Association/National Heart, Lung, and Blood Institute Scientific Statement: [1] increased waist circumference (>102 cm for man and >88 cm for woman), [2] elevated triglycerides (≥150 mg/dL) or use of triglyceride-lowering drugs, [3] low levels of high-density lipoprotein cholesterol (HDL-C) (<40 mg/dL in men and <50 mg/dL in women), [4] hypertension (≥130/85 mmHg) or use of antihypertensive drugs, and [5] elevated fasting glucose (≥100 mg/dL) or drug treatment for elevated blood glucose [9].

### 2.3. Anthropometric Characteristics

Waist circumference (WC) was assessed at the end of consecutive natural breaths, midpoint between the top of the iliac crest and the lower margin of the last palpable rib in the midaxillary line, and at a level parallel to the floor. The cutoff points of abdominal obesity were defined according to National Cholesterol Education Program Adult Treatment Panel III (WC > 88 cm for women and WC > 102 cm for men) [9]. Weight status was defined by body mass index (BMI) (normal weight: 18.5–24.9 kg/m^2^; overweight: 25–29.9 kg/m^2^, obese: ≥30 kg/m^2^) [10].

### 2.4. Laboratory Analysis

Fasting plasma samples were obtained from study participants after an overnight fasting on first day of hospitalization. Serum cholesterol, triglycerides (TG), HDL-C, and low-density lipoprotein cholesterol (LDL-C) were measured by enzymatic colorimetric method with commercially available kit (COBAS 311, Roche Diagnostics GmbH, Mannheim, Germany). Serum concentrations of glucose were determined enzymatically using the hexokinase method (Roche Diagnostics GmbH, Mannheim, Germany). The particle-enhanced immunoturbidimetric method with Behring BN-100 Nephelometer (Behring Diagnostics, Frankfurt, Germany) was used to measure C-reactive protein (CRP). Serum levels of aspartate aminotransferase (AST), alanine aminotransferase (ALT), gamma-glutamyl transferase (GGT), creatinine, and uric acid were assessed by using a commercial biochemistry analyzer (Roche p800 Modular, Roche Diagnostics, Indianapolis, IN, USA).

### 2.5. Thorax CT Assessment

CT imaging was performed in all hospitalized patients with suspected COVID-19 pneumonia. A semiquantitative CT severity scoring suggested by RSNA [11] was used for interpretation of severity of radiological involvement. CT severity score (CTSS) was calculated separately for 6 lung zones as follows: 1, <0–25% involvement; 2, 25–50% involvement; 3, 50–75% involvement; and 4, 75–100% involvement. The overall CTSS was calculated as the sum of the individual zonal scores and maximum score was 24. According to the RSNA report, 1–6 points: mild involvement, 7–11 points: moderate involvement, and >12 points: severe involvement [11].

### 2.6. Statistical Analysis

Frequency and percentage values were given for categorical variables. Mean, standard deviation, median, minimum, and maximum values were given for continuous variables. The normal distribution test of continuous variables was performed with the Kolmogorov–Smirnov test. Chi-squared analysis was used for the relationships between categorical variables. Where appropriate, categorical variables were evaluated with Fisher's exact test. Independent sample *t*-test was used to compare two groups in continuous independent variables with normal distribution. The Mann–Whitney *U* test was used in the comparison of two independent groups for the variables that did not fulfill the assumption of normal distribution. Logistic regression analysis was used to determine the effect of independent variables on disease severity. *p* < 0.05 was considered statistically significant. Analysis of data used the program SPSS Statistics 23.0 (IBM Corporation, Armonk, New York, USA).

## 3. Results

The study population consisted of 60 (36 male and 24 female) patients with moderate COVID-19 disease patients and 30 (24 male and 6 female) severe COVID-19 disease patients. Comparative analysis of metabolic syndrome and associated components according to COVID-19 disease severity are summarized in Table 1. 83.33% of patients with severe COVID-19 disease have MetS. However, in patients with moderate COVID-19 disease, MetS was seen with a rate of 58.33% (*p*=0.018). Among the MetS criteria, elevated TG and the presence of hyperglycemia were significantly higher in severe COVID-19 patients (*p*=0.035 and *p*=0.010, respectively). On the other hand, abdominal obesity, presence of low HDL-C levels, and hypertension did not differ between two groups (*p* > 0.05 for all). In addition, while T2DM was seen in 50% of the severe cases (*p*=0.002), CAD was found in similar percentages in moderate and severe COVID-19 cases (*p*=0.204).

According to the laboratory tests performed on admission, fasting blood glucose (FBG) and HbA1c values were higher in patients with severe COVID-19 disease (*p*=0.004 and *p*=0.007) (Table 1). While serum D-dimer, ferritin, fibrinogen, and CRP values were similar in moderate and severe COVID-19 disease (*p* > 0.05 for all), the mean serum procalcitonin was higher in severe cases (*p*=0.033). The severe COVID-19 group had significantly lower lymphocyte count compared with moderate COVID-19 disease (1.02 ± 0.39 vs 1.79 ± 3.89, *p*=0.016) (Table 1).

Table 2 shows the clinical parameters of patients with (*n* = 60) and without MetS (*n* = 30). The number of patients with severe COVID-19 disease was more prominent in patients with MetS compared with patients without MetS (41.6% vs 16.6%, *p* < 0.001) (Table 2). The percentage of male patients was higher in MetS patients than in non-MetS patients (*p*=0.018). As expected, patients with MetS have higher BMI (*p* < 0.001), waist circumference (*p* < 0.001), systolic blood pressure (*p*=0.038), diastolic blood pressure (*p*=0.002), FBG (*p* < 0.001), HbA1c (*p* < 0.001), and triglycerides (*p* < 0.001) than non-MetS group. In addition, serum HDL-C was lower in MetS group (*p* < 0.001). Serum ferritin, D-dimer, LDH, and fibrinogen levels were similar in both patients with and without MetS (*p* > 0.05). On the other hand, the mean CRP value was 83.86 ± 72.42 in MetS patients and 50.23 ± 86.34 in non-MetS patients (*p*=0.002) (Table 2). There was no significant difference in length of hospitalization between MetS group and non-MetS group (*p*=0.492). In the MetS group, 8.5% of the patients needed ICU admittance, 5% needed mechanical ventilation, and 3.3% died.

COVID-19 pneumonia severity was assessed according to computerized tomography severity score (CTSS), which was found higher in patients with MetS than in patients with non-MetS (*p* < 0.001). Based on CTSS, rate of severe involvement was observed higher in patients with MetS (*p* < 0.001) (Table 2).

A logistic regression model was created in which the parameters related to MetS components were the independent variables and the severity of the disease was the dependent variable. Out of components of MetS, serum triglycerides (*p*=0.024), waist circumference (*p*=0.004), and serum HDL-C (*p*=0.006) had an effect on the COVID-19 severity (Table 3).

Hospital treatments and previous medications of patients with COVID-19 disease are represented in Supplementary Table 1. Favipiravir and enoxaparin were the standard regimens and taken by all patients. Although the percentage of patients with severe COVID-19 disease needing 6 mg dexamethasone treatment was higher, the difference between the groups was statistically insignificant (*p*=0.06). However, it was observed that pulse methylprednisolone therapy was needed higher rates in patients with severe COVID-19 disease (*p*=0.001).

## 4. Discussion

To date, observational studies demonstrated that obesity, hypertension, and diabetes are risk factors for severe course of COVID-19 disease and associated with fatal outcomes [12]. Based on the fact that abdominal obesity, hypertension, and diabetes are the components of MetS, we aimed to study the MetS components and severe course of COVID-19 disease association. Current study suggests that serum triglycerides, serum HDL-C levels, and waist circumference are risk factors for severe course in patients with COVID-19 disease.

The pathophysiological background of the association between impaired metabolic health and COVID-19 has been investigating extensively. One of the blamed pathways is the interaction of SARS-CoV-2 and angiotensin-converting enzyme (ACE-2), which is expressed in respiratory epithelial cells, pancreatic islets, vascular endothelium, and adipose tissue [13]. ACE-2 protein expression is predicted to be affected by glucose and lipid metabolism. Rosiglitazone upregulates vascular ACE-2 protein expression in hypertensive rats [14]. Also, atorvastatin and fluvastatin increase cardiac ACE-2 protein expression in rats [15]. Another mechanism is the direct invasion of SARS-CoV-2 in endothelial cells, resulting in endothelial inflammation in vital organs and vascular endothelial system [16]. MetS presumed to cause chronic endothelial dysfunction may exacerbate endotheliitis in COVID-19 disease [17]. Our study demonstrated that patients with severe COVID-19 have higher prevalence of MetS. The findings of our study are consistent with the previous studies. In a multicenter retrospective study, cardiometabolic disorder has been linked to mortality and severity of COVID-19 infection [18]. Another study of 287 patients with COVID-19 demonstrated increased mortality in patients with MetS [19]. Similarly, a recent large-scale study demonstrated higher prevalence of MetS in severe COVID-19 than in mild-to-moderate COVID-19 disease. It was revealed that particularly central obesity, impaired fasting glucose, and hypertriglyceridemia were associated with the disease severity [20]. We suggest a possible link between hypertriglyceridemia and severe COVID-19 disease among MetS components. A recent striking retrospective study showed that hypertriglyceridemia in patients with COVID-19 disease increases mortality by 2.3-fold, independent of diabetes and obesity [21]. Limited hypothesis exists to explain increased serum triglyceride levels in COVID-19 infection. Firstly, during infectious state, lipoprotein lipase (LPL)-mediated blood triglyceride clearance decreases, which may induce hypertriglyceridemia [22,23]. Secondly, serum triglyceride itself may promote inflammation by activating leukocytes, increasing extravasation of monocytes, and accumulation of tissue macrophages [24,25]. Together with the findings of our findings, it can be predicted that serum triglyceride elevation may directly contribute to clinical deterioration in COVID-19 pneumonia.

HDL-C itself is suggested to be involved in the regulation of innate immune response through interaction with ABCA1 or ABCG1, which negatively regulates T-cell activation and the expression of inflammatory mediators [26]. In addition, decreased number of small HDL particles is inversely associated with the disease activity score and CRP levels during inflammatory states [27]. Studies during COVID-19 pandemic showed reduced HDL-C levels reflecting reduced levels of HDL particles in plasma of severe COVID-19 patients [28,29]. We demonstrated low HDL-C levels are related to severity of COVID-19 in accordance with the recent literature.

Obesity-mediated inflammatory response is another factor contributes to the unfavorable prognosis in obese COVID-19 patients. Obese patients have higher concentration of pro-inflammatory cytokines such as TNF-a, IL-6, and MCP-1 [30]. Also, a recent meta-analysis has highlighted the vulnerability of obese patients for COVID-19 infection and the importance of the management of weight management [31]. Report from the USA showed that among COVID-19 patients, those with BMI>30 kg/m2 were 1.8 to 3.6 times more likely to be admitted to ICU compared with those with BMI <30 kg/m2 [32]. Chronic activation of the immune system, increased levels of circulating chemokines and adipokines, and variation in pulmonary mechanics resulting in altered topographic distribution of ventilation are possible explanations for unfavorable outcomes in COVID-19 patients with obesity [31,33]. In Turkey, limited meta-analysis has been performed on the prevalence of obesity. One of them showed that the prevalence of obesity was 33.2% in men and 18.2% in women [34]. Due to the high prevalence of obesity and its association with severe COVID-19 disease, it draws particular attention to the impact of obesity on disease severity in this geographic region. In this single-center prospective study, patients with severe and moderate COVID-19 disease were found to have similar BMI values below 30 kg/m2. Sahin et al. showed that obese patients with BMI ≥35 kg/m2 had higher lung involvement rates and required longer noninvasive mechanical ventilation than overweight patients [35]. While several studies evaluated the impact of obesity on COVID-19 morbidity and mortality, the clinical course of overweight patients and various degrees of obesity have not been involved in meta-analysis.

Although similar BMI values in moderate and severe COVID-19 cases, we have demonstrated that WC has an impact on COVID-19 disease severity. It has been suggested that visceral adipose tissue is blamed for proinflammatory state in obesity. WC and WHR are the anthropometric measurements that reflect visceral adipose tissue accumulation and abdominal obesity [36]. Limited studies during COVID-19 pandemic have demonstrated conflicting results regarding the effect of obesity phenotypes and disease severity. Khalangot et al. found that body mass index determined obesity had no effect on mortality, but WC was an independent risk factor for mortality in hospitalized patients with COVID-19 disease [37]. However, Freuer et al. suggested that the impact of obesity is weaker than BMI on the susceptibility and severity of COVID-19 disease [38]. Further studies are required to clarify the underlying mechanism linking visceral adipose tissue and obesity with COVID-19 infection.

Available data on hypertension and COVID-19 morbidity and mortality suggest conflicting results. Several studies have reported the link between poor blood pressure control and the risk of developing severe COVID-19 due to activated renin-angiotensin-aldosterone system as well as resulting in a procoagulant and inflammatory response [39,40]. By contrast, in patients with MetS, higher blood pressure has been found inversely associated with the mortality in COVID-19 [20]. In this present study, both hypertension prevalence on admission and antihypertensive drug treatment history were similar in moderate and severe COVID-19 disease. In addition to antihypertensive drug usage history, severe and moderate COVID-19 patients have similar treatment histories regarding both diabetes and hyperlipidemia. We observed that patients with severe COVID-19 had higher HbA1c and FBG in addition to the increased prevalence of diabetes. Despite similar history of antidiabetic treatment, the increased prevalence of diabetes may the result of inadequate treatment. Furthermore, we observed increased fasting blood glucose was observed at admission in severe COVID-19 pneumonia. A recent study of patients with COVID-19 suggested that fasting plasma glucose ≥126 mg/dl is an independent factor for mortality [41]. In a retrospective cohort study with COVID-19 disease, diabetics had shorter overall survival times than nondiabetics [42]. On the other hand, among patients with diabetes, uncontrolled glycemic control related to a higher mortality in respect to controlled diabetes [43]. In addition, Alguwaihes et al. demonstrated that DM patients have a significantly higher death rate and lower survival time than non-DM patients [44]. Possible pathogenetic mechanisms underlying diabetes and COVID-19 disease are deteriorated immune function, inflammation and glucotoxicity, activation of renin-angiotensin-aldosterone system, and endothelial damage [45]. In Turkey Diabetes Epidemiology Study (TURDEP I and II), it was observed that glucose tolerance increased by 106% and diabetes increased by 90% in ten years. Diabetes proportion of those who have not been diagnosed before was 45.5% [46]. This means that one in a two people with diabetes is undiagnosed, as in the world. Therefore, in the COVID-19 pandemic, diabetes has a special importance in terms as a risk factor for increased mortality and morbidity.

It is known that patients with severe course of COVID-19 have increased levels of inflammation-related biomarkers and are closely associated with poor prognosis [47]. We analyzed radiological data as well as laboratory findings and found that patients with MetS had a significantly higher RSNA-recommended CT severity score. These results indicate that patients with MetS had more severe inflammatory response and lung infiltration than non-MetS population.

This study has several limitations. First, the small sample size of SARS-CoV-2 patients with MetS may have affected the statistical comparison of MetS components in the course of severe COVID-19 infection. In addition, patients with critical and mild COVID-19 disease could not be evaluated because the study was conducted on COVID-19 patients hospitalized in the ward. In particular, the role of MetS components in critical course of COVID-19 disease is one of the key points in the assessment of unfavorable end points. As this study reflects the findings of the second wave, comparative studies among first, second, third, and fourth waves of pandemic will enable to investigating the effects of different treatment strategies in COVID-19 infection.

## 5. Conclusion

In this pilot study, 83.33% of patients hospitalized with severe COVID-19 disease had MetS. Among MetS components, serum triglycerides, serum HDL-C, and waist circumference are associated with the severe course of COVID-19 disease. Therefore, the findings of the present study highlight the importance of the burden of MetS in COVID-19 disease.

## Figures and Tables

**Table 1:** Patients' characteristics of moderate and severe COVID-19 patients.

	Moderate COVID-19 (*n* = 60)	Severe COVID-19 (*n* = 30)	*p*-value
	N (%)	N (%)	
Demographic variables			
Age (mean ± SD)	50.82 ± 10.51	50.47 ± 9.87	0.901
Sex (male), n (%)	36 (60)	24 (80)	0.097
BMI (kg/m^2^) (mean ± SD)	29.16 ± 4.2	29.69 ± 5.18	0.854
Metabolic syndrome	35 (58.33)	25 (83.33)	0.018
Abdominal obesity	33 (55)	17 (56.66)	0.881
Hypertension	26 (43.33)	13 (43.33)	1.000
Elevated TG	30 (50)	22 (73.33)	0.035
Low-HDL-C	48 (80)	21 (70)	0.225
Hyperglycemia	29 (48.33)	23 (76.66)	0.010
T2DM	16 (26.66)	15 (50)	0.002
CAD	2 (3.33)	3 (10)	0.204

*Measurements*	*mean* *±* *SD*	*mean* *±* *SD*	
WC (cm)	98.47 ± 12.01	101.37 ± 10.79	0.348
SBP (mmHg)	121.58 ± 18.5	122.1 ± 10.58	0.429
DBP (mmHg)	73.08 ± 13.09	74.8 ± 7.82	0.134
FBG (mg/dl)	114.03 ± 48.36	145.72 ± 64.07	0.004
HbA1c (%)	6.37 ± 0.99	7.55 ± 2.21	0.007
T. Cholesterol (mg/dl)	161.52 ± 39.55	165.72 ± 37.13	0.534
Triglycerides (mg/dl)	171.55 ± 93.75	217.11 ± 90.9	0.007
LDL-C (mg/dl)	92.57 ± 29.31	101.44 ± 30.68	0.249
HDL-C (mg/dl)	33.84 ± 9.32	35.8 ± 9.7	0.263
Creatinine (mg/dl)	0.88 ± 0.3	0.93 ± 0.29	0.399
CRP (mg/L)	67.91 ± 83.57	82.72 ± 67.18	0.142
Lymphocytes (x10^9^/L)	1.79 ± 3.89	1.02 ± 0.39	0.016
Ferritin (ng/ml)	570.31 ± 508.79	577.16 ± 503.35	0.906
D-dimer (*μ*g/ml)	1.38 ± 1.96	1.02 ± 0.93	0.546
LDH (mg/dl)	346.35 ± 122.39	413.47 ± 190.92	0.191
Procalcitonin (ng/ml)	0.11 ± 0.13	0.15 ± 0.11	0.033
Fibrinogen (g/L)	590.42 ± 129.5	581.47 ± 137.36	0.590

Statistical significance at *p* < 0.05. BMI: body mass index, MetS: metabolic syndrome, WC: waist circumference, FBG: fasting blood glucose, SBP: systolic blood pressure, and HDL-C: high-density lipoprotein cholesterol.

**Table 2:** Clinical, laboratory, and radiological characteristics of patients with COVID-19 disease according to the presence of metabolic syndrome.

	MetS	Non-MetS	*p*-value
	*n* = 60	*n* = 30	
	mean ± SD	mean ± SD	
Age (years)	51.72 ± 10.09	48.67 ± 10.41	0.229
Sex (male), n(%)	45 (75)	15 (50)	0.018
BMI (kg/m^2^)	30.8 ± 4.37	26.41 ± 3.29	<0.001
WC (cm)	103.93 ± 10.36	90.43 ± 8.47	<0.001
Systolic BP (mmHg)	124.12 ± 17.77	117.03 ± 11.48	0.038
Diastolic BP (mmHg)	76.25 ± 12.29	68.47 ± 7.91	0.002
FBG (mg/dl)	138.23 ± 61.6	98.13 ± 27.95	<0.001
Triglycerides (mg/dl)	210.54 ± 96.7	139.12 ± 70.95	<0.001
HDL-C (mg/dl)	31.55 ± 8.59	40.3 ± 8.39	<0.001
Lymphocytes (x10^9^/L)	1.61 ± 3.90	1.37 ± 0.67	0.049
Procalcitonin (ng/ml)	0.14 ± 0.14	0.09 ± 0.07	0.050
Ferritin (ng/ml)	588.11 ± 478.38	536.27 ± 568.4	0.209
D-dimer (*μ*g/ml)	1.25 ± 1.78	1.26 ± 1.52	0.575
LDH (mg/dl)	381.28 ± 149.68	343.3 ± 156.37	0.186
Fibrinogen (g/L)	596.65 ± 129.25	569 ± 136.12	0.350
CRP (mg/L)	83.86 ± 72.42	50.23 ± 86.34	0.002
Severe COVID-19 cases, n(%)	25 (41.6)	5 (16.6)	0.018
ICU, n(%)	5 (8.3)	0	0.104
MV, n(%)	3 (5)	0	0.213
Mortality, n(%)	2 (3.3)	0	0.312
Length of hospitalization (days)	9.02 ± 4.55	8.34 ± 3.97	0.492
Chest CTSS	13.88 ± 5.9	8.10 ± 3.05	<0.001
Lung involvement, n(%)			
Mild involvement	10 (6)	12 (36.6)	<0.001
Moderate involvement	16 (26.6)	15 (50)	
Severe involvement	34 (56.6)	4 (13.3)	

Statistical significance at *p* < 0.05. BMI: body mass index, BP: blood pressure, FBG: fasting blood glucose, T. Cholesterol: total cholesterol, HDL-C: high-density lipoprotein cholesterol, LDH: lactate dehydrogenase, CRP: C-reactive protein, ICU: intensive care unit, MV: mechanical ventilation, and CTSS: computerized tomography severity score.

**Table 3:** The effect of metabolic syndrome parameters on COVID-19 disease severity

	B	*p*-value	O.R	95% C.I.for O.R	
				Lower	Upper
FBG	0.017	0.128	1.017	1.000	1.012
Triglycerides	0.014	0.024	1.014	1.002	1.027
HDL-C	−0.180	0.006	0.835	0.735	0.949
Waist circumference	0.174	0.004	1.190	1.057	1.339
Systolic BP	0.018	0.563	1.018	0.958	1.082
Diastolic BP	0.034	0.525	1.035	0.931	1.151

Statistical significance at *p* < 0.05. FBG: fasting blood glucose, HDL-C: high-density lipoprotein cholesterol, and BP: blood pressure.

## Data Availability

Data are available upon request from the corresponding author.

## Supplementary Materials

Supplementary Materials Supplementary Table 1. In-hospital treatments and previous medications of patients with COVID-19 disease. ()
