# Supplementary material for: Relationship between Metabolic Syndrome Components and COVID-19 Disease Severity in Hospitalized Patients: A Pilot Study
Source: Can J Infect Dis Med Microbiol. 2022 Aug 24;2022:9682032. doi: 10.1155/2022/9682032 (PMC9433267; doi:10.1155/2022/9682032)
Supplement: Supplementary Materials — Supplementary Table 1. In-hospital treatments and previous medications of patients with COVID-19 disease. () [file 9682032.f1.docx]

Supplementary Table-1: In hospital treatments and previous medications of patients with COVID-19 disease

|  | Moderate COVID-19  *n*=60, n(%) | Severe COVID-19  *n*=30, n(%) | *p*-value |
| --- | --- | --- | --- |
| **In hospital treatment** |  |  |  |
| Antibiotics | 21 (61.76) | 13 (38.24) | 0.442 |
| Favipiravir | 60(100) | 30(100) | 1.000 |
| Enoxaparin | 60(100) | 30(100) | 1.000 |
| 6mg Dexamethasone | 41 (68.33) | 26 (86.66) | 0.060 |
| Pulse Methylprednisolone | 2 (3.33) | 11 (36.66) | 0.001 |
| Colchicine | 32(53.33) | 17(56.66) | 1.000 |
| **Previous medications** |  |  |  |
| RAS blockers | 9(69,23) | 4(30,77) | 0.807 |
| Beta blockers | 9(15) | 1(3.33) | 0.155 |
| CCB | 7(11.66) | 3(10) | 0.813 |
| Diuretics | 3(5) | 1(3.33) | 1.000 |
| OAD | 7(11.66) | 5(8.33) | 0.529 |
| Insulin | 3(5) | 3(10) | 0.396 |
| Lipid lowering drugs | 5(8.33) | 0(0) | 0.165 |
| Antiaggregant | 4(6.66) | 4(13.33) | 0.433 |

Statistical significance at p<0.05. Abbreviations: CCB: calcium canal blockers, OAD: oral antidiabetics.
